# Transcriptional basis of the acclimation to high environmental temperature at the olfactory receptor organs of *Drosophila melanogaster*

**DOI:** 10.1186/1471-2164-14-259

**Published:** 2013-04-17

**Authors:** Jacob Riveron, Tamara Boto, Esther Alcorta

**Affiliations:** 1 Department of Functional Biology, Faculty of Medicine, University of Oviedo, Oviedo, 33006, Spain; 2 Present address: Vector Group, Liverpool School of Tropical Medicine, Pembroke PI, Liverpool, Merseyside, L3 5QA, UK; 3 Present address: Department of Neuroscience, The Scripps Research Institute, Jupiter, Florida, 33458, USA

**Keywords:** Olfaction, Olfactory acclimation, Olfactory reception, Environmental temperature, *Drosophila melanogaster*, Microarray analysis, Olfactory reception genes

## Abstract

**Background:**

Environmental temperature directly affects the concentrations of chemicals in the gas phase. Therefore, if the olfactory system does not physiologically adapt to environmental conditions, it may provide inadequate information about the distance to or direction of odor sources. Previous reports have shown at the behavioral level that temperature induces changes in olfactory sensitivity in *Drosophila melanogaster*. These changes are initiated in the main olfactory receptor organs, the antennae. In this work, we attempted to identify the particular genes responsible for olfactory adaptation to increasing temperatures in these organs based on current knowledge of the molecular basis of olfactory reception.

**Results:**

Whole-genome transcriptional responses to transitory temperature shifts from 21-30°C were analyzed in the third antennal segments of *Drosophila*. More than 53% of the genome was expressed in these organs; this percentage increased slightly (55%) after heat treatment. However, the expression levels increased for 26%, decreased for 21% and remained constant for 53% of the expressed genes. Analysis of the changes produced in 389 genes related to heat response and olfactory reception, according to the current functional annotations of the *Drosophila* gene set, showed significant differences in 95 of these genes, which are involved in the heat response (23), perireceptor events in olfaction (50), olfactory and gustatory receptors (18) and G-proteins and transduction cascades (4).

**Conclusions:**

Gene expression was altered in response to environmental heat in the antennae of *Drosophila* by increasing or decreasing expression. Different acclimation patterns emerged for reception through the basiconic, trichoid and coeloconic sensilla. Changes in genes with a central role in olfactory reception, such as *orco*, may account for part of the acclimation reported at the behavioral level.

## Background

Organisms live in a dynamic environment in which climatic factors change continuously, not only between different seasons but also during the same day or in the different microhabitats in a small area. In this continuously changing environment, sensory systems in general and the olfactory system in particular must be able to adjust to provide accurate information to organisms [1].

The environmental temperature affects the concentrations of chemicals in the gas phase. Thus, rising temperatures increase the volatility and concentrations of odorants in the air. Several reports have addressed the ability of the olfactory system to adapt to high odorant concentrations in the environment [1]. In *Drosophila*, central [2,3] and peripheral elements of the olfactory system are responsible for such adaptation [4,5].

Furthermore, it has been shown through behavioral tests that temperature influences olfactory sensitivity in *Drosophila melanogaster*[6]. The biological effects of olfactory adaptation to temperatures fluctuating within the normal range, from 15 to 30°C, have been reported. At intermediate odorant concentrations, the environmental temperature and olfactory sensitivity appear to be negatively correlated. These results support the notion that adaptation of the olfactory system provides accurate information to the animal, compensating for modifications of chemical volatility. Further studies involving the main olfactory receptor organs, the antennae, have shown that changes in olfactory responses begin during the reception process and appear at the neuron receptor level [7].

The same pattern of responses has been observed whether the temperature shift lasts a few hours or several days. Therefore, searching for gene expression changes, which are most likely necessary for long-lasting responses, may help to shed light on the basis of olfactory adaptation to heat at the reception level. To elucidate the molecular mechanisms involved in this process, we performed an analysis of differential gene expression in the antennae using microarrays.

Whole-genome gene expression arrays have been used previously in *Drosophila* to identify genes that are responsible for adaptation to high and low temperatures. For example, gene expression patterns have been analyzed in the following contexts: a) selection experiments for heat and cold resistance [8,9]; b) *Drosophila* lines subjected to different heat treatments [10]; and c) natural populations corresponding to different geographical locations [11]. However, in these studies, emphasis was placed on global issues concerning the effect of heat stress on the whole organism and not on the particular response of the olfactory system.

Some attention has also been paid to the changes in the transcriptional profiles of olfactory genes under different biological conditions [12] and in response to special treatments. Due to the social impact of alcoholism, several microarray studies have focused on understanding the molecular changes that occur after exposure to ethanol using various model organisms [13]. Thus, it is known that in *D. melanogaster*, exposure to ethanol causes a decrease in the expression of genes affecting olfaction, among other changes [14-17].

Other studies related to olfactory gene expression have focused on insect vectors of human diseases, such as the malaria mosquito, *Anopheles gambiae*[18-21]. Thus, the differential expression of odorant-binding–protein genes (OBPs) has been found to be related to the eating behavior of male and female mosquitoes, depending on their metabolic status [22,23].

In this report, we concentrate on the genome expression changes that appear in the main olfactory receptor organs of *Drosophila* after exposure to high temperatures. With this aim, wild-type Canton-S flies were subjected to 48-hour treatments at 30°C. First, we provide a general overview of the genes whose expression is most altered due to heat, based on the Gene Ontology (GO) functional groups defined in *Drosophila*. Then, to examine the processes related to the olfactory function in greater detail, we used a direct approach, selecting specific tissues and conducting a priori selection of the genes to be examined via microarray analysis using literature-based functional information. Differential gene expression analyses of the third antennal segments were performed to compare control and treated flies.

Special attention was paid to those genes related to olfactory reception and heat response, which were represented by 389 probesets. These genes may play an important role in the olfactory adaptation of insects to heat, which has been demonstrated functionally at the whole-individual level [6] and in the olfactory receptor organs [7].

Finally, we tested the contribution to the adaptation to heat of the *orco* gene, which is a gene related to olfactory reception that is expressed in more than 70% of olfactory receptor neurons [24]. With this goal, we simulated the expression changes in this gene due to heat via genetic manipulation and studied the functional consequences in response to odor.

## Results and discussion

### RT-PCR validation

The microarray results were validated via real time-PCR for 9 genes, representing approximately 10% of the genes selected based on their potential interest from the larger pool of genes demonstrating significant changes in expression in the microarray analysis (95/389). *βtubulin60D* was used as an internal control. An equal efficiency for every pair of primers compared to the controls was confirmed, and the fold-change levels were determined. The results were consistent with the microarray analysis data with respect to the direction and amount of change, 5 of which were up-regulated, while 4 were down-regulated (Table 1). Regression analysis of the qPCR fold-change levels compared to the correspondent microarray results for the 9 genes yielded the following regression line y = 0.775× + 0.206 with a highly significant correlation value of r^2^ = 0.999 (Ftest = 6641.86, P < 0.0001).

**Table 1:** Expression changes due to the heat treatment measured using microarrays or RT-PCR

**Gene**	**Fold change**	**Fold change**
	**(Microarrays)**	**(RT-PCR)**
*or47b*	1.66	1.33
*gr21a*	1.67	2.09
*hsp67Bb*	32.54	25.43
*per*	0.51	0.71
*hsc70Cb*	1.92	1.31
*ir21a*	1.42	1.30
*ugt86Da*	0.76	0.81
*pbrp4*	0.71	0.61
*obp49a*	0.68	0.72

### Microarray analysis

**Figure 1:**
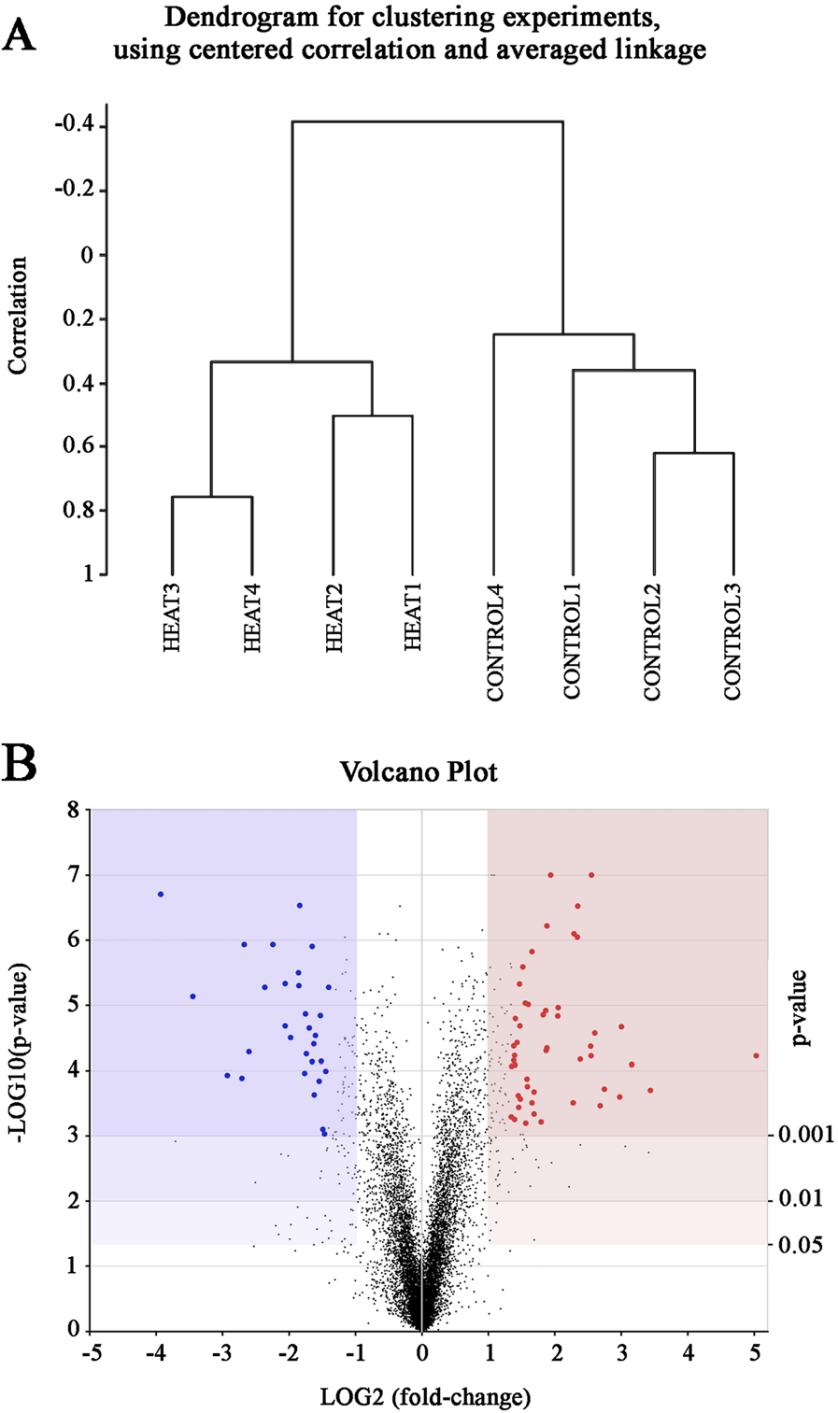
**General microarray analysis. A**) Dendrogram for clustering experiments using centered correlation and averaged linkage. The data from the 8 microarrays were included: 4 under control conditions and 4 after heat treatment. **B**) Volcano plot of the microarray results. Light blue region: significantly decreased expression, indicated a reduction of 50%. Light red: region of increased expression, indicating a 2-fold increase in gene expression compared to control. Three levels of significance are indicated by tone. Red and blue points represent the 80 genes that showed the largest changes in expression (10% of the genes that change significantly at P < 0.001) under the heat treatment.

A total of 8 microarrays were analyzed, 4 for each group of control or heat-treated flies. Pools of approximately 4,000 antennae per array were used (see Methods). Prior to the gene expression analysis, the samples were paired in a dendrogram based on a comparison of the expression profiles of all the genes (Figure 1A). The antennae samples collected from heat-treated flies formed a separate and distant cluster from the control samples, which aggregated in a second cluster. This observation reflects the effectiveness of the treatment and the importance of the temperature changes in the regulation of gene expression.

Figure 1B provides a volcano plot of the microarray results. Both increased and decreased gene expression changes occurred due to heat treatment. The expression values that decreased by less than 1/2 compared with the treatment are included in the blue area, while increases of more than two fold are shown in the light red region. In both regions, the genes that exhibited the greatest changes in expression due to the heat treatment have been marked with colored dots; these genes will be described below.

Next, we focused on three separate issues: a) ascertaining whether a given gene was expressed in the antennae in the experimental and control groups; b) obtaining a general overview of the genes whose expression was changed most due to the treatment and identifying the functional groups to which they belong; and c) determining the differences in the mean expression of particular groups of genes (olfactory and thermal stress-related) between the heat-treated and the control groups.

We note that a gene was considered to be expressed in the antennae in each group when it appeared as “Present” in the statistical detection calls for the four replicates in the group provided by the Affymetrix GeneChip® microarray analysis software (see the RNA extraction and microarrays subsection in the Methods section).

The raw microarray data have been deposited in the following public database: ArrayExpress at the European Bioinformatics Institute (EBI), http://www.ebi.ac.uk/arrayexpress; Accession number: E-MTAB-1228.

#### Total gene expression in the third antennal segment in Drosophila

**Figure 2:**
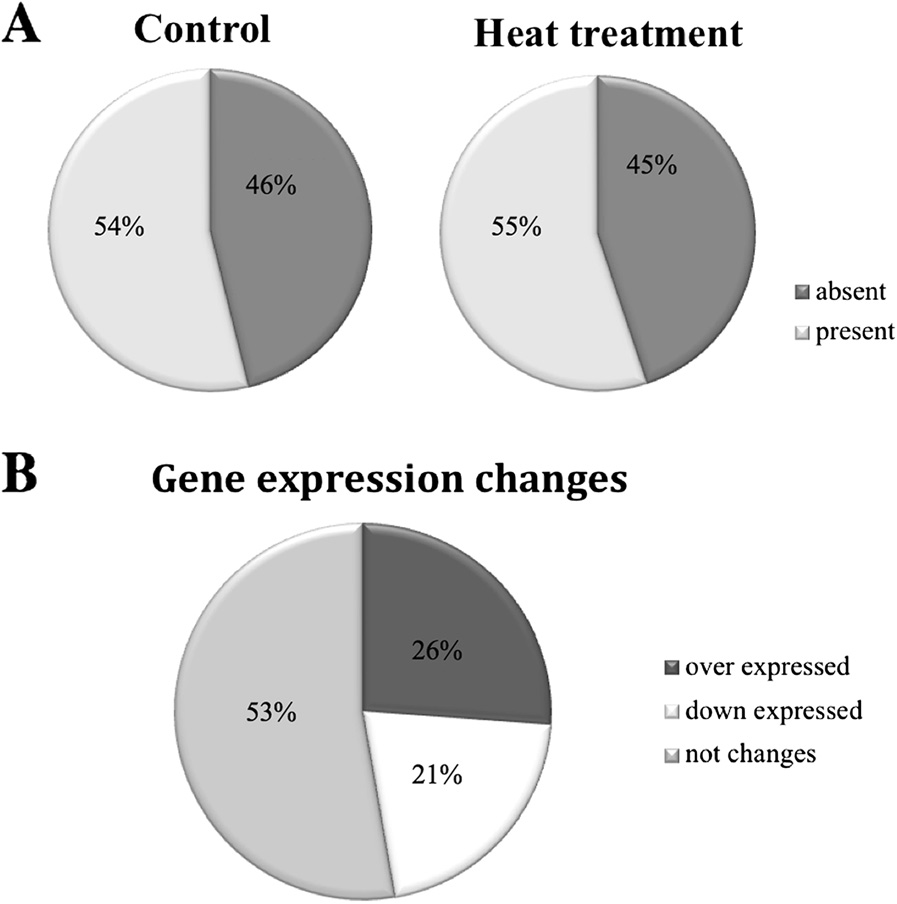
**Gene expression in the third antennal segment of *Drosophila melanogaster*. A**) Percentage of probe sets that show expression under the control and heat treatment conditions among the total genome. **B**) Global effects of the heat treatment in antennae, measured as the percentage of probe sets that showed expression.

The third antennal segments are the main olfactory receptor organs in *Drosophila*. These segments express genes related to olfactory reception as well as other genes with more general functions. As a first approach, we studied how many genes were expressed in the antennae. Of the 14,445 probe sets encoding a transcript in *Drosophila*, 7,774 (54%) were expressed in the control group, while in the experimental group subjected to the heat treatment, 7,957 (55%) were “Present” (Figure 2A). More than half of the genome was expressed in the *Drosophila* third antennal segment, showing the same range of gene expression (54%-63%) observed in the chemosensory appendages and even in other tissues (bodies) in *Anopheles gambiae*[25].

Based on the total values obtained, it appeared that heat might induce an increase of gene expression. To test this hypothesis, we analyzed the changes in gene expression levels due to heat treatment (Figure 2B). A total of 3,846 probe sets showed a change in expression because of the treatment (FDR < 0.1), among which 2,135 (26%) increased, and 1,711 (21%) decreased. Again, the overall expression levels appeared to be higher in the heat-treated than in the control group. However, both groups showed both increased and decreased gene expression after the heat treatment, thereby excluding the possibility of a general rule relating increased gene expression to temperature increases.

#### Functional GO groups most affected by heat treatment

To obtain an idea of the main functional processes affected by heat, we analyzed the genes whose expression showed the greatest changes following heat treatment. We studied a 10% of the genes that change significantly at P < 0.001. They undergo fold changes > 2.6 including both increases and decreases in expression (Additional file 1: Table S1) for a total of 80 genes. As shown by the colored dots in Figure 1B, increased expression was observed for approximately 2/3 of the genes, while a decrease was observed for approximately 1/3.

**Figure 3:**
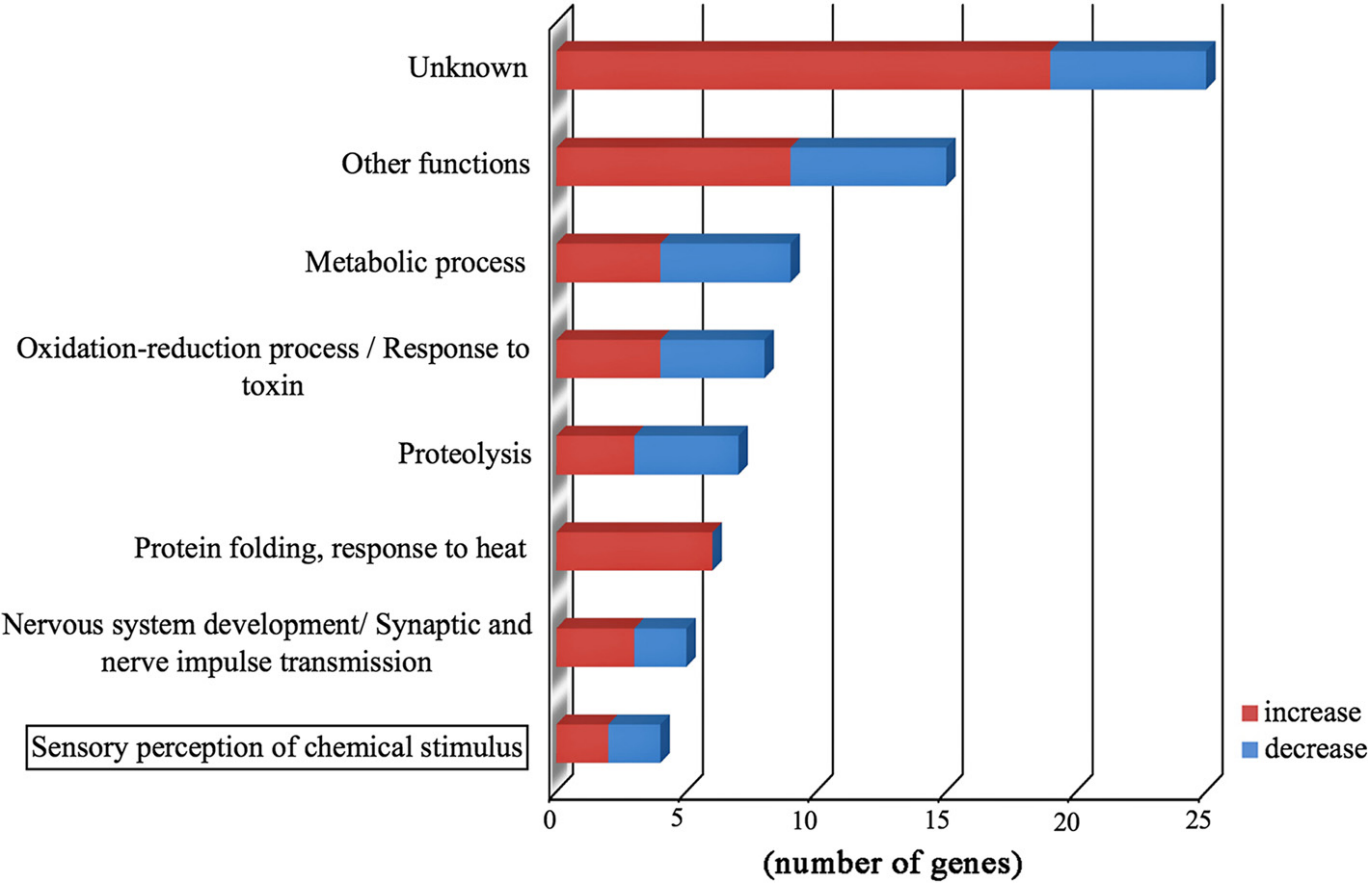
Gene Ontology (GO) groups based on biological process for the 80 genes that showed the greatest changes in expression under heat treatment.

These data are summarized in Figure 3 based on functional groups of GO biological process (information in GO molecular function and cellular component for each gene is also presented in Additional file 1: Table S1), distinguishing genes showing increased or decreased expression using different colors. Approximately 1/3 of these genes have unknown functions. The other defined groups were represented by 4–9 genes each, with no obvious over-representation observed. The residual, heterogeneous group included 15 genes. As expected under heat treatment, many genes from the HSP family (6 genes), which are associated with the response to heat, were found on this list and were always over-expressed. The gene that presented the largest expression change was Hsp67b (fold change > 32), followed by Hsp70Ab (> 10).

Additionally, some genes related to metabolic processes were present on the list (9 genes), but not all of these genes were overexpressed, indicating that both activation and inhibition of metabolic processes are important in the response to heat.

The detected genes corresponding to the sensory perception of chemical stimuli mainly encoded odorant-binding proteins (4 genes). In the group of oxidation-reduction processes/responses to toxins, there were several genes previously associated with other perireceptor processes in olfactory reception, such as genes encoding members of the Cyp family (4 genes) and Glutathione-S-transferase. However, all of these groups included genes that were greatly over-expressed or strongly inhibited, and none of them showed a clear pattern of expression (Additional file 1: Table S1).

#### Expression of olfactory and thermal stress genes

To obtain a greater understanding of the molecular basis of the adaptation of olfactory reception to temperature and considering that small expression changes in key genes could affect olfaction beyond quantitative expectations, we studied particular groups of genes that encode elements that may affect “a priori” this process. A total of 389 genes were selected based on the literature (because in most cases, each gene was represented by a single probe set, the terms gene and probe set will be used interchangeably in the text). The complete gene list and the original results are included in Additional file 2: Table S2. Table 2 summarizes the results obtained for each of the following groups: a) genes related to thermal stress, including those encoding heat shock proteins (HSPs), cold shock domain proteins (CSDPs) and other thermal stress-related genes; and b) genes related to olfactory reception, such as those encoding odorant binding proteins (OBPS), cytochrome P-450 mono-oxygenases (referred to as CYPs or P450 enzymes), UDP-Glucuronosyl transferases (UGTs), glutathione-S-transferases (GSTs), olfactory receptors (ORs and IRs), taste receptors (GRs), G-proteins, members of the cAMP signal transduction cascade and components of the IP3 pathway.

**Table 2:** Summary of the changes induced by the heat treatment in a group of 389 probe sets corresponding to a) thermal stress and b) olfactory reception genes

	**Number of genes showing expression differences at FDR < 0.1**				
	**Gene class**	**Present/analyzed**	**Total changes with heat (% of present)**	**Over-expression (% of present)**	**Down-expression (% of present)**
**Thermal stress**	**HSPs**	18/26	15 (83%)	15 (83%)	0 (0%)
	**CSDPs**	3/4	1 (33%)	0 (0%)	1 (33%)
	**Other thermal genes**	15/27	7 (47%)	2 (13%)	5 (33%)
**Olfactory reception**	**OBPs**	19/47	11 (58%)	7 (37%)	4 (21%)
	**PBPRPs**	6/6	3 (50%)	0 (0%)	3 (50%)
	**CYPs**	54/84	24 (44%)	10 (18%)	14 (25%)
	**UGTs**	10/18	3 (30%)	0 (0%)	3 (30%)
	**GSTs**	17/20	9 (53%)	5 (29%)	4 (24%)
	**ORs**	38/40	9 (24%)	4 (11%)	5 (13%)
	**IRs**	13/16	4 (31%)	4 (31%)	0 (0%)
	**GRs**	11/66	5 (45%)	3 (27%)	2 (18%)
	**G-PROTEINS**	13/14	2 (15%)	0 (0%)	2 (15%)
	**AMPc pathway**	8/9	1 (12%)	1 (12%)	0 (0%)
	**IP3 pathway**	10/12	1 (10%)	1 (10%)	0 (0%)
	**TOTAL**	235/389	95 (40%)	52 (22%)	43 (18%)

Among the 389 probe sets that were analyzed, 235 showed expression in at least the four samples from the experimental or the control groups. Of these 235 genes, a total of 95 exhibited modified expression in response to heat treatment, either presenting increased (52) or decreased (43) expression. In 8 out of the 14 groups, the changes within the group were coincident in their direction in all of the significant cases. However, for some groups, very few genes showed significant expression changes; this observation is described in further detail below.

#### Genes related to thermal responses

Several groups of genes have been associated with the response to different temperatures. They are related to the following products: heat shock proteins (HSPs); cold shock domain proteins (CSDPs), which are similar to those observed in the cold shock proteins (CSPs) of bacteria; and other genes related to the thermo-protection function.

In our study, we decided to include these genes as an internal control of appropriate heat treatment application in the experimental group because it is known that, especially for *hsps*, their expression levels increase with temperature [26-28].

In general, approximately half of the probe sets encoding products of response to heat stress showed increased expression, although there was also a significant fraction, approximately 17%, that showed decreased expression. These data confirmed that the response to heat stress is mediated not only by the overexpression of certain thermo-protectors genes but also by the inhibition of the expression of others.

As for the different groups we studied, it should be noted that virtually all of the genes encoding HSPs present in the tissue displayed increased expression (15 out of 18 probesets) in individuals that were subjected to heat shock (Table 3). These results were expected if we consider the role this group of genes plays in protection against heat shock. Specifically, *hsp83* and *DnaJ-1* (also known as *hsp40*), which showed significantly increased expression in the experimental group, have been related to the protection of synaptic transmission [29]. According to these results, the heat treatment was correctly applied to the flies.

**Table 3:** Thermal stress genes

**Probe set ID**	**p value**	**FDR**	**Gene symbol**	**Expression level (Control)**	**Expression level (Heat)**	**Fold-change**	**Direction of change**
**HSPs**							
1638872_at	1.00E-07	3.90E-05	*hsp68*	9.46	36.49	3.86	↑
1641055_at	2.66E-05	1.15E-03	*hsp23*	17.01	103.22	6.07	↑
1629061_s_at	6.03E-05	1.73E-03	*hsp67Bb/hsp22*	7.58	246.51	32.54	↑
1630487_s_at	8.13E-05	1.87E-03	*hsp67Bb/hsp22*	5.43	48.74	8.98	↑
1639571_s_at	2.01E-04	3.47E-03	*hsp70Ab/hsp70Aa*	12.20	133.08	10.91	↑
1628117_at	2.03E-04	3.47E-03	*hsp27*	19.15	43.02	2.25	↑
1635044_at	3.41E-04	4.93E-03	*hsp26*	8.88	57.04	6.42	↑
1641286_s_at	7.26E-04	9.14E-03	*hsp60*	634.29	1272.85	2.01	↑
1636741_s_at	1.32E-03	1.32E-02	*hsc70Cb*	168.96	324.72	1.92	↑
1626821_s_at	1.45E-03	1.35E-02	*hsp70Ba/Bb/Bbb/Bc*	12.03	100.23	8.33	↑
1637059_s_at	5.34E-03	3.23E-02	*dnaJ-1*	1212.11	2323.72	1.92	↑
1632841_x_at	6.04E-03	3.37E-02	*hsp70Bc*	7.80	36.24	4.65	↑
1630637_s_at	1.30E-02	5.97E-02	*hsc70-4*	6263.29	8913.44	1.42	↑
1638484_at	1.42E-02	6.35E-02	*hsp67Bc*	4.91	7.03	1.43	↑
1630688_at	2.37E-02	9.51E-02	*hsp83*	2870.70	5068.79	1.77	↑
**CSDPs**							
1639908_a_at	3.30E-03	2.22E-02	*CG9705*	309.24	248.56	0.80	↓
**OTHER THERMAL GENES**							
1638937_at	1.49E-03	1.35E-02	*anon-23Da*	1998.45	1277.18	0.64	↓
1635450_a_at	1.72E-03	1.52E-02	*smp-30*	440.83	358.12	0.81	↓
1631333_s_at	2.31E-03	1.84E-02	*adh*	212.45	699.39	3.29	↑
1638452_at	3.55E-03	2.31E-02	*per*	61.97	31.74	0.51	↓
1627458_at	5.72E-03	3.24E-02	*catsup*	330.68	259.13	0.78	↓
1625949_at	2.04E-02	8.75E-02	*gpdh*	250.19	382.42	1.53	↑
1634893_at	2.33E-02	9.48E-02	*gpdh*	1118.48	989.07	0.88	↓

Probe sets showing altered transcriptional regulation after heat treatment at FDR < 0.1.

As for the 4 genes of the CSDPs group, 3 of them, *unr*, *yps* and CG9705, are present on the antennae of adult individuals, but only CG9705 showed significant differences, decreasing its expression after applying the heat shock. We observed opposite expression behaviors of the heat and cold shock protein-encoding genes in response to temperature treatments, as was expected.

Finally, 7 other genes related to thermo-protection functions changed their expression significantly. However, they belong to a heterogeneous group, and the expression of these genes either increased or decreased. In this group, we include the genes *anon-23 Da*, *smp-30*, *per*, *catsup*, *adh* and two different transcripts of the *gpdh* gene, Fbtr0079147 and Fbtr0079146 (transcript annotation in Flybase), which displayed opposite expression behaviors in response to heat. Down regulation of *per* gene expression can be related to a previous study that reported delayed expression of the morning oscillation peak of *per* at high temperatures [30].

#### Genes encoding components of perireceptor events

The so-called perireceptor events in olfactory reception occur in the lymph of the olfactory sensilla [31]. Odorant binding proteins (OBPS), Cytochrome P-450 mono-oxygenases (CYPs), UDP-Glucuronosyl transferases (UGTs) and glutathione-S-transferases (GSTs) are proteins that have been associated with these processes. In addition, some proteins related to the detection of pheromones that also function as OBPS have been described in other *Drosophila* species, which have been designated PBPRPs (Pheromone Binding Proteins-Related Proteins) and LUSH. We will also describe the analysis of these groups (Table 4).

**Table 4:** Olfactory reception genes: perireceptor events

**Probe Set ID**	**p value**	**FDR**	**Gene symbol**	**Expression level (Control)**	**Expression level (Heat)**	**Fold-change**	**Direction of change**
**OBPs**							
1638374_at	8.00E-07	1.56E-04	*obp19b*	71.09	349.57	4.92	↑
1625531_at	1.37E-05	1.01E-03	*obp18a*	85.63	25.49	0.30	↓
1641288_at	6.56E-05	1.73E-03	*obp57b*	372.95	541.16	1.45	↑
1626586_at	1.12E-04	2.42E-03	*obp57a*	213.04	62.81	0.30	↓
1638276_at	2.57E-04	4.00E-03	*obp56e*	31.87	252.05	7.91	↑
1623659_at	8.67E-04	1.04E-02	*obp56d*	8517.02	14521.22	1.71	↑
1624074_at	1.49E-03	1.35E-02	*obp56a*	8.87	26.56	2.99	↑
1639597_at	1.80E-03	1.56E-02	*obp44a*	20.04	213.35	10.65	↑
1624932_at	2.90E-03	2.05E-02	*obp49a*	323.15	218.07	0.68	↓
1634121_at	2.97E-03	2.07E-02	*obp57c*	1362.99	1686.91	1.24	↑
1637914_at	3.06E-03	2.09E-02	*obp83g*	48.67	22.39	0.46	↓
1630963_at	1.68E-04	3.28E-03	*pbprp4*	7327.95	5218.26	0.71	↓
1641200_at	5.46E-03	3.23E-02	*pbprp3*	22781.68	20750.76	0.91	↓
1626882_at	1.03E-02	5.16E-02	*lush*	9726.46	8670.25	0.89	↓
**CYPs**							
1634731_at	9.70E-06	1.01E-03	*cyp4p3*	26.01	78.50	3.02	↑
1641428_at	1.08E-05	1.01E-03	*cyp9c1*	21.51	89.31	4.15	↑
1636292_at	2.07E-05	1.01E-03	*cyp313a4*	246.70	59.27	0.24	↓
1640566_at	2.07E-05	1.01E-03	*cyp4p2*	6.68	14.72	2.21	↑
1634640_at	3.31E-05	1.29E-03	*cyp4ac1*	41.66	18.62	0.45	↓
1634662_at	5.99E-05	1.73E-03	*cyp313a1*	106.14	619.88	5.84	↑
1630170_at	2.05E-04	3.47E-03	*cyp12b2*	97.66	72.44	0.74	↓
1638562_a_at	2.43E-04	3.95E-03	*cyp6d5*	2273.70	1238.88	0.55	↓
1633639_at	9.84E-04	1.07E-02	*cyp28d1*	33.61	18.42	0.55	↓
1629610_at	1.29E-03	1.32E-02	*cyp28c1*	481.27	236.01	0.49	↓
1632114_at	1.38E-03	1.35E-02	*cyp12a4*	208.42	136.22	0.65	↓
1637309_a_at	1.88E-03	1.59E-02	*cyp12e1*	39.93	58.32	1.46	↑
1640755_at	2.05E-03	1.66E-02	*cyp6a8*	4012.95	1794.62	0.45	↓
1639495_at	2.58E-03	1.94E-02	*cyp9b1*	49.32	191.03	3.87	↑
1634143_at	2.63E-03	1.94E-02	*cyp6w1*	10481.51	7490.12	0.72	↓
1626198_at	2.85E-03	2.05E-02	*cyp4d8*	765.77	539.45	0.70	↓
1629009_at	3.43E-03	2.27E-02	*cyp28a5*	573.81	479.64	0.84	↓
1623068_at	5.40E-03	3.23E-02	*cyp4e3*	215.36	153.79	0.71	↓
1638053_at	5.69E-03	3.24E-02	*cyp4p1*	314.86	382.49	1.22	↑
1635008_at	7.34E-03	3.92E-02	*cyp9b2*	193.78	373.71	1.93	↑
1639539_at	8.62E-03	4.54E-02	*cyp4e1*	197.84	137.04	0.69	↓
1623866_at	9.54E-03	4.96E-02	*cyp4ac2*	683.94	428.93	0.63	↓
1627974_at	1.20E-02	5.70E-02	*cyp6t3*	1226.10	2090.66	1.71	↑
1624101_at	2.07E-02	8.76E-02	*cyp6a23*	49.59	70.99	1.43	↑
**UGTs**							
1624465_at	7.97E-05	1.87E-03	*ugt*	163.99	101.28	0.62	↓
1627662_at	4.10E-04	5.71E-03	*ugt35a*	49.23	20.63	0.42	↓
1624156_at	1.93E-02	8.35E-02	*ugt86Da*	857.99	650.80	0.76	↓
**GSTs**							
1627890_at	5.59E-05	1.73E-03	*gstD10*	57.17	17.23	0.30	↓
1624732_at	5.50E-04	7.15E-03	*gstE5*	900.79	1707.24	1.90	↑
1623957_s_at	1.22E-03	1.28E-02	*gstS1*	415.66	258.03	0.62	↓
1626136_at	1.91E-03	1.59E-02	*gstD6*	8.86	5.64	0.64	↓
1636174_at	2.36E-03	1.84E-02	*gstD9*	146.47	239.80	1.64	↑
1635701_at	5.67E-03	3.24E-02	*gstD3*	80.44	137.18	1.71	↑
1634554_at	7.15E-03	3.87E-02	*gstD8*	482.07	677.21	1.41	↑
1625744_at	1.32E-02	5.97E-02	*gstE6*	1670.99	2452.98	1.47	↑
1637129_at	2.09E-02	8.78E-02	*gstE3*	1333.61	1054.64	0.79	↓

Probe sets showing altered transcriptional regulation after heat treatment at FDR < 0.1.

Following the heat treatment, 47% (50/106) of the probe sets related to proteins involved in perireceptor processes occurring in the antennae showed a change in gene expression. In 21% of the cases, gene expression increased, and in 26%, it decreased.

Approximately 58% (11/19) of the *Obps* genes present in the antenna presented a change in expression after heat treatment, although there was no single consistent pattern of change observed. Expression increased for 7 genes (*obp19b, obp57b, obp56e, obp56d, obp56a, obp44a, obp57c*) and decreased for 4 (*obp18a, obp57a, obp49a, obp83g*). Attempts to classify the OBPs based on the specific odorants whose reception they mediate failed to clarify these results. It has been reported that OBPs function in a combinatorial manner [32], and there is some odorant overlap in the profiles of the following OBPs: a) obp18a and obp56a and b) 57a and 57b [33], which showed opposite expression responses to heat.

Among the genes involved in pheromone detection at the perireceptor level, the intensity values for the corresponding probe sets were extremely high, even under control conditions. In this study, there were 3 genes that showed significantly decreased expression after the heat treatment: *pbprp4*, *pbprp3* and *lush*, the last two of which are expressed in the lymph of the trichoid sensilla [32].

The expression levels of other genes in this group also appeared to be reduced by heat treatment, although these differences were not significant. This reduction was in agreement with the changes reported in response to high concentrations of ethanol [14,17].

There was a total of 24 genes encoding CYPs that showed altered expression in response to heat treatment (44%), with expression increasing significantly for 10 of these genes and decreasing for 14.

Of the genes encoding UGTs, 3 genes, *ugt*, *ugt35a* and *ugt86Da*, exhibited a small, but significant decrease in expression when subjected to heat shock, while the rest of the UGTs remained unchanged.

Finally, when we analyzed the GSTs, 5 genes, *gstE5*, *gstD9*, *gstD3*, *gstE6* and *gstD8*, were found to show significantly increased expression when individuals were subjected to 48 hours of heat treatment, while 4 genes, *gstD10*, *gstS1*, *gstE3* and *gstD6*, presented decreased expression. Increases in the expression of *gst* genes have been related to the development of chemical resistance in many insect species and mammalian systems [34] as well as in the development of alcohol preferences in rats [13,34,35].

#### Genes encoding molecular receptors

Until 2009, the ORs were the only olfactory receptors that had been described in *Drosophila*[36,37]. However, another 3 taste receptors present in the antennae have now been reported to bind to odor molecules [38-40]: GR10a, GR21a and GR63a. In 2009, a new family of genes that encode ionotropic receptors, the IRS, was reported to function as molecular receptors involved in smell in *Drosophila*[41], and their olfactory profile was described [42]. In the present study, we analyzed all members of the three gene families described in antennae: the *or, ir* and *gr* gene families.

After heat shock treatment, 29% of the probe sets for olfactory receptors showed a change in intensity (18% increased, while 11% decreased) (Table 5).

**Table 5:** Olfactory reception genes: receptors

**Probe Set ID**	**p value**	**FDR**	**Gene symbol**	**Expression level (Control)**	**Expression level (Heat)**	**Fold-change**	**Direction of change**
**ORs**							
1637114_at	5.29E-04	7.12E-03	*or47b*	231.09	379.88	1.64	↑
1631298_at	8.79E-04	1.04E-02	*or22b*	508.23	204.64	0.40	↓
1638928_at	7.12E-03	3.87E-02	*or23a*	82.61	70.27	0.85	↓
1629154_at	1.03E-02	5.16E-02	*or43a*	63.42	88.06	1.39	↑
1636134_at	1.07E-02	5.26E-02	*or83b*	5033.00	3735.63	0.74	↓
1627819_at	1.10E-02	5.37E-02	*or13a*	10.90	19.34	1.78	↑
1639973_a_at	1.24E-02	5.75E-02	*or69a*	24.90	18.72	0.75	↓
1638902_at	1.51E-02	6.68E-02	*or47a*	195.12	163.63	0.84	↓
1631562_at	2.16E-02	8.94E-02	*or88a*	84.62	124.17	1.47	↑
**IRs**							
1633636_at	3.11E-04	4.67E-03	*ir64a*	280.42	452.47	1.61	↑
1633365_at	2.60E-03	1.94E-02	*ir76b*	144.09	218.73	1.52	↑
1633880_s_at	1.02E-02	5.16E-02	*ir76a*	107.47	139.77	1.30	↑
1631795_at	1.24E-02	5.75E-02	*ir21a*	28.74	40.92	1.42	↑
**GRs**							
1629589_at	1.56E-05	1.01E-03	*gr43a*	27.93	13.88	0.50	↓
1629590_at	9.78E-04	1.07E-02	*gr64c*	83.08	32.84	0.40	↓
1631563_at	4.25E-03	2.72E-02	*gr21a*	39.53	65.82	1.67	↑
1638594_at	1.15E-02	5.56E-02	*gr28b*	15.88	20.39	1.28	↑
1640888_a_at	2.39E-02	9.51E-02	*gr64e*	48.53	59.62	1.23	↑

Probe sets showing altered transcriptional regulation after heat treatment at FDR < 0.1.

If we separate the observations for all of the antennal *or* genes, only *or98b*, which corresponds to the ab6B type ORN (olfactory receptor neuron), did not appear in any of the 8 arrays used here (Additional file 2: Table S2). Additionally, *or2a*, of the at3 ORN, was not found in some of the arrays and was therefore considered absent. The absence of these receptors in our samples was most likely due to the techniques and methods that were used here not being sufficiently sensitive to detect their messenger RNAs.

Among the *or* members present in our samples, 4 genes, *or47b*, *or43a*, *or13a* and *or88a*, showed significantly increased expression under heat treatment, while 5 of them, *or22b*, *or23a*, *or69a*, *or47a* and co-receptor *or83b* (now called *orco*) [43], presented decreased expression.

Although it initially appeared that there was no unique pattern of change regarding the expression of *or* genes in response to high temperatures, a common profile emerged when we considered the individual *or* members: 3 out of the 4 non-general *or* genes showing significantly decreased expression due to heat shock (*or22b*, *or69a* and *or47a*) corresponded to basiconic sensilla (ab3, ab9 and ab5, respectively), while the ORs exhibiting increased expression (*or47b*, *or43a*, *or88a* and *13a*) corresponded to trichoid or intermediate sensilla (at4, at3, at4 and ai1). However, the correlation was not complete, and receptor *or23a*, which showed down-regulation under heat, was expressed in trichoid sensillum at2.

On the other hand, down-regulation of the expression of a single gene, the common co-receptor *orco*, could control the number of ORs present in the membrane of the ORNs, according to its proposed role in directing receptor migration inside the cell [24]. Interestingly, a reduction of the level of *orco* expression has been reported in response to high concentrations of ethanol [14]. Because increasing temperature enhances the odorant concentration in the gas phase, these results may suggest that the co-receptor expression level may be involved in adaptation to high odorant concentrations.

Regarding the *ir* genes, we detected all of the previously described genes in the antenna, except *ir93a*. Of the 13 probe sets that were detected, 4 genes, *ir64a*, *ir76b*, *ir76a*, *ir21a*, showed significantly increased expression after heat shock, representing 30.77% of the total. A common pattern of increased gene expression in response to heat can be established for these genes. *ir76b* encodes the co-receptor of the ORNs of the four types of coeloconic sensilla, ac1-ac4, and may therefore influence odorant sensitivity in all of them. The gene products of *ir64a* and *ir76a* have been related to other co-receptors, IR8a in the sacculus and IR25a in sensilla ac4, respectively. Finally, *ir21a* has been reported to show expression at the arista and perhaps sacculus III. It has been noted that aristal neurons function as thermosensors [44], but the role of IR21a, if any, in mediating physiological responses to temperature changes, is unknown [42].

Finally, of the 66 probe sets related to *gr* genes that were examined, only 11 were present in the antennal tissue; these included the three that were previously described: *gr10a*, *gr21a* and *gr63a*. Among these genes, only *gr21a*, which is related to CO2 reception [39,40], showed a significant increase in expression associated with heat, while the other two exhibited no change. Of the other 8 probe sets present in the antenna, 4 showed a significant change in expression when *Drosophila* were subjected to heat shock, either increasing, as observed for *gr28b* and *gr64e,* or decreasing, as observed for *gr43a* and *gr64c*.

#### Genes related to transduction cascades

Although the direct involvement of transduction cascades in olfactory reception is still under discussion in *Drosophila*, some behavioral data suggest a role for the cAMP and DAG/IP3 pathways [45-47]. Therefore, we included some of the genes that mediate these processes in our analyses, such as genes encoding G-proteins and others related to either the cAMP or the DAG/IP3 cascade (Table 6).

**Table 6:** Olfactory reception genes: transduction cascades

**Probe Set ID**	**p value**	**FDR**	**Gene symbol**	**Expression level (Control)**	**Expression level (Heat)**	**Fold-change**	**Direction of change**
**G proteins**							
1637526_s_at	6.65E-05	1.73E-03	*Ggamma30A*	94.79	45.06	0.48	↓
1633439_at	1.49E-04	3.06E-03	*Galpha73B*	213.15	94.02	0.44	↓
**AMPc pathway**							
1635928_a_at	5.25E-03	3.23E-02	*pka-C3*	274.00	487.64	1.78	↑
**IP3 pathway**							
1626289_at	1.60E-02	7.03E-02	*dgkepsilon*	142.18	170.57	1.20	↑

Probe sets showing altered transcriptional regulation after heat treatment at FDR < 0.1.

The results of the analysis of the presence of G-proteins in antennae were in agreement with those reported in previous studies [48]. All of the genes encoding the alpha, beta and gamma subunits were expressed in the antenna, except for Gbeta76c and some transcripts of Galpha49B, a variant of Gqalpha. Two of these genes showed a significant change in expression following heat shock: *Ggamma30A* and *Galpha73B* (*Gf*). There were two probe sets encoding the first gene, one representing all known transcripts of the gene (Fbtr0079795, Fbtr0079796, Fbtr0079797) and another that encodes only one (Fbtr009797). Because the latter probe displayed low intensity values, indicating a lack of or very weak expression, the observed differences in gene expression must have been due to the action of the other transcripts (Fbtr0079795 and/or Fbtr0079796). Both *Ggamma30A* and *Galpha73B* showed decreased expression in the heat-treated group compared to control group.

Among the 9 genes in the cAMP pathway that were studied, only one gene encoding a protein kinase, *pka-C3*, showed a significantly higher intensity in the probe set after the heat shock treatment.

Finally, of the 12 probe sets related to the DAG/IP3 transduction cascade that were examined, *dgkepsilon* was the only gene that showed a significant difference, presenting increased expression in the heat-treated group compared to control group. The role of the *dgkepsilon* gene product also involves protein kinase activity. It has been proposed that the balance of phosphorylation is often critical for regulating enzyme function, mediating protein–protein interactions, altering the subcellular localization of proteins and controlling protein stability. Furthermore, kinases and phosphatases may work together to modulate the strength of a signal [49]. However, it must be kept in mind that transduction cascades mediate many cellular processes.

### Behavioral consequences of the changes in *orco* gene expression

The microarray analysis identified a set of genes related to heat shock as well as olfaction that showed altered expression in response to temperature increases. To determine whether the expression changes observed for some of these genes can account for a portion of the previously described olfactory acclimation [6,7], we performed additional experiments.

**Figure 4:**
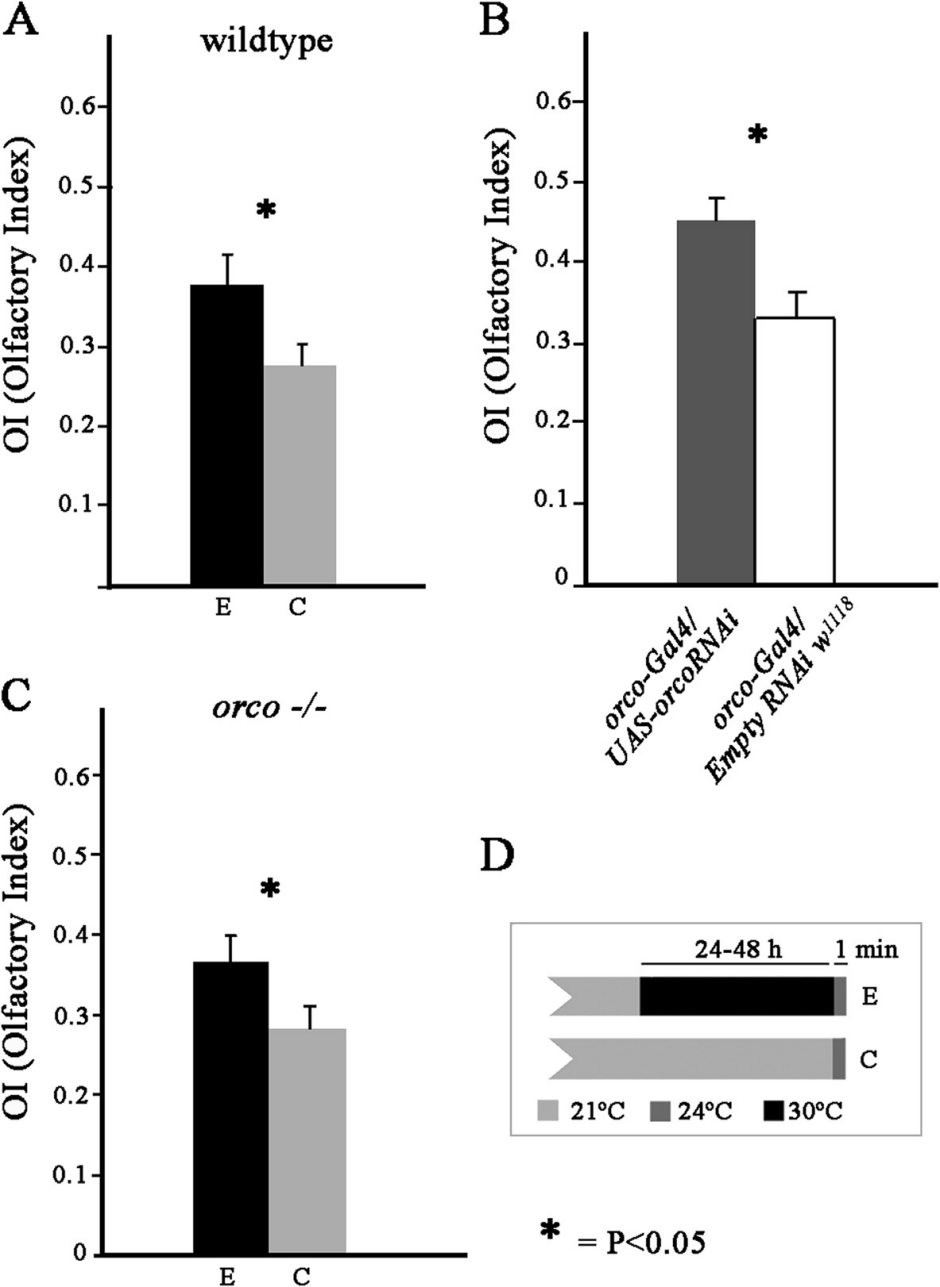
**Changes in the olfactory preference in the T-maze due to heat. A**) The repellent responses to 10^-1.5^ vol/vol ethanol in water are reduced in wildtype flies due to the heat treatment [IO = number flies on odor side/(number flies on odor + control sides); IO > 0.5 indicates attraction, IO = 0.5 indicates indifference, and 0 < IO < 0.5 indicates repellency). **B**) Olfactory responses to 10^-1.5^ ethanol in flies with reduced levels of the Orco co-receptor compared with control flies of the same genetic background. **C**) The repellent responses to 10^-0.5^ ethanol are reduced in *orco* null mutants due to the heat treatment. **D**) Temperature protocol for the control C and experimental E flies shown in panels **A**) and **C**). Note that the behavioral response was measured for both groups at the same temperature and therefore at the same odorant concentration in the gas phase.

First, we chose a single gene that may affect olfaction in a generalized manner. This type of gene is very difficult to find at the receptor level because of the combinatorial coding of olfactory information, which utilizes many olfactory receptors with differential specificities to capture a single odor. The *orco* gene encodes a co-receptor that dimerizes with the ORs and is expressed in more than 70% of ORNs [50]. Next, we attempted to simulate the change in expression that was produced by the heat treatment (a decrease for the *orco* gene) via genetic manipulation using RNAi and studied the behavioral consequences on olfaction (Figure 4B). Heat-induced olfactory sensitivity changes that were especially apparent at intermediate repellent concentrations were noted as a partial sensitivity loss [6].

Using a T-maze, we compared the response to 10^-1.5^ vol/vol ethanol in water (the concentration that evoked intermediate repellent responses in the control group) of flies showing reduced expression of *orco* (*orco-Gal4/UAS-orcoRNAi* heterozygotes) with the corresponding control (*orco-Gal4/line 6000*) (Figure 4B). The repellent response was reduced in the experimental hybrids (t = 2.527, nE = 34, nC = 39, P = 0.0137*), as would be expected according to the results of the microarray analysis. This suggests that the conclusions of previous experiments showing that null *orco* mutants reduced very significantly the response to odorants [50] could be also applied to our experimental conditions by affecting the *orco* expression level. In the complete absence of the *orco* gene product, the Orco co-receptor, olfactory defects are observed because of the inability of the specific ORs to localize to the dendritic surface of ORNs [24,50]. It is possible that altering the *orco* expression level may control ORN sensitivity in a quantitative manner by affecting the number of ORs that locate in the dendritic surface.

To explore the possibility that adaptation may occur in ORNs that express molecular receptors other than ORs, such as IRs, we studied the *orco* null mutant under normal conditions and after 24 hours of heat-shock treatment (previous experiments [6] showed that 6–48 h of heat shock have the same behavioral consequences, but we chose this treatment period here because it allows flies to retain a better condition regarding behavioral performance than under 48 h treatments).

ORNs containing IRs appear to be less responsive to ethanol than those containing ORs [42]. Therefore, we had to block the responses through OR-dependent ORNs to observe IR dependent behavior.In this case, we increased the odorant concentration to 10^-0.5^ vol/vol ethanol in water to find the intermediate repellent response region in the control condition (with no heat-shock treatment). The repellent responses to ethanol were reduced after heat shock (Figure 4C) in the same direction as previously described for normal flies after temperature acclimation. The differences were statistically significant: t = 2.074, nE = 32, nC = 33, P = 0.0422*. It therefore appears that the adaptation to temperature is not only dependent on the *orco* gene. The higher concentration required to achieve intermediate repellent responses and differences observed under heat shock may indicate that the *orco* null mutant exhibits reduced sensitivity compared to the wildtype control (as shown in Figure 4B) and that the other elements that mediate adaptation in this experiment made a relatively small quantitative contribution. However, we cannot exclude the possibility that differences in genetic background may play a role.

All of these results are consistent with the current understanding of olfactory reception. Ethanol is detected by the ORNs of the basiconic [51] and coeloconic sensilla [42], but with different specificities and effects. It has been reported that the overall responses to 10^-2^ vol/vol ethanol in ORNs with ORs are excitatory, whereas the responses in ORNs with IRs appear to be inhibitory and less intense [42].

Thus, adaptation during the response to ethanol together with increasing temperatures could be mediated to a large extent by the basiconic sensilla, which show reduced expression of olfactory receptors, *orco* and particularly *or* genes. To a lesser extent, the coeloconic sensilla would adapt by increasing the expression of *ir* genes (such as *ir76a*, which encodes the co-receptor for all the ORNs expressing IRs) that play an inhibitory role, as was shown in the microarray analysis.

## Conclusions

The sense of smell adapts to changes in the environmental temperature via the adjustment of sensitivity in a direction that compensates for changes in the concentrations of odorous compounds in the gas phase due to their change in volatility [6]. Acclimation is initiated in the olfactory receptor organs [7]. To describe the mechanisms responsible for such adaptation, we performed a microarray analysis of the main olfactory receptor organs (the third antennal segments) of flies subjected to high temperatures compared with controls not subjected to temperature treatment.

Significant changes in the expression of genes related to the responses to heat and stress, such as increases in many genes in the heat shock protein family (HSPs) and decreases of some cold shock domain proteins (CSDPs), confirmed the efficiency of the applied treatment in the antennal tissue.

We also observed changes in gene expression concerning olfactory reception. The observation of genes related to perireceptor events, molecular olfactory receptors and transduction cascades before and after heat shock allowed us to glimpse certain gene expression patterns that might at least partially explain the observed functional changes.

It has been proposed that in *Drosophila*, olfactory reception takes place through several different routes [42,52], which we will attempt to relate to our results. The basiconic, trichoid and coeloconic sensilla constitute a complex reception system, with each type of sensilla presenting its own characteristics according to its structure, the type of olfactory receptors expressed in its ORNs and the odorant type that it is able to detect.

General odor reception is performed in the basiconic sensilla and is mediated by ORs, which dimerize with the co-receptor Orco. The decreased sensitivity that has been reported at the behavioral level after heat shock could be associated with a decrease in the number of molecular receptors in the membranes of ORNs. In this work, we found that among the *or* genes whose expression was changed significantly after temperature treatment, there was a decreased response when they correspond to the ORNs of basiconic sensilla. Decreased expression has also been found for the ubiquitous Orco co-receptor, which is necessary for the transport and insertion of odorant receptors in the chemosensory dendritic membranes of olfactory neurons [50]. Interestingly, adaptation to high levels of ethanol has also been related to acute down-regulation of *orco* and other olfactory genes [13-15,17].

The only OBP that has been clearly localized to the basiconic sensilla is PBPR5 [53], and it showed no significant differences in expression due to heat.

In the trichoid sensilla, which mediate the response to pheromones in some cases [54], down-regulation of the expression of the specific odorant binding proteins LUSH and PBPRP3 has been found to be associated with increased temperatures or high concentrations of ethanol [13-15,17]. Therefore, in this type of sensilla, the control mechanism causing the system to become less sensitive may begin with a change in the solubility of pheromones in the lymph surrounding the ORNs. Among the ORs expressed in the ORNs of trichoid sensilla, we observed that when they showed a change in expression under heat treatment, it was in the form of an increase (with one exception being observed, for an OR of at2). This change in expression appears to occur in the opposite direction compared to the basiconic sensilla. However, these ORs preferably show inhibitory responses in empty neurons [51], indicating either that they mediate actual inhibitory responses or that they function in a different way. Thus, for the at1 sensilla, an inhibitory-excitatory relationship has been suggested between LUSH and SNMP and the Or67d/Orco elements of this system. Moreover, different responses have been recorded when Or67d has been expressed in trichoid or basiconic sensilla [52]. In the present study, neither *SNMP* nor *or67d* showed a significant change in expression, but decreases in LUSH and Orco could control the entire system.

The coeloconic sensilla contain ORNs that express different types of olfactory receptors, the Irs. They play a role that is complementary to olfaction through the ORs [42]. Whether the response of the Ir system is to specific or common compounds, it preferentially displays inhibitory responses. Interestingly, microarray analysis found some differences in the gene expression response to heat, always in the direction of up-regulation. One of the genes that exhibited a change in expression has been described as the co-receptor for ORNs containing Irs. In this case, an increased number of molecular receptors inducing more inhibitory responses might confer the same result as the decrease of the excitation of basiconic sensilla.

There are also a few changes related to transduction cascade genes that could be contributing to olfactory modulation in the acclimation to high temperatures.

Achieving a better understanding of the basic mechanisms of olfactory reception in the future may help to explain the differences observed in other genes related to perireceptor events; in most cases, these genes failed to show a uniform pattern. For example, the genes encoding CYPs and GSTs showed both up- and down-regulation, and among the UGTs, there were only 3 genes presenting significantly decreased expression.

## Methods

### Drosophila stocks and heat treatment

The standard Canton-S line of *Drosophila melanogaster*, obtained from the Bloomington Stock Center (BSC, Indiana, USA), was used in the experiments. The flies were bred at 21±1°C, with light/dark cycles of 12:12 hours in 220 cc. bottles with a common yeast-sugar-agar medium. In the experimental group, 2-to-8-day-old flies were maintained for 48 h at 30°C±1°C. For the control group, the flies remained at the initial growing temperature. Before the treatment experimental and control flies were transferred to new bottles with a near-odorless medium composed of 5 g/l agarose, 50 g/l sucrose and water. This measure was adopted in an attempt to minimize the differential effects of environmental odor in the experimental group versus the control.

For the behavioral validation of the effects of the *or83b* (*orco*) gene, the following stocks were used: a natural population (P2), that exhibits the same olfactory phenotype in response to heat as the standard wildtype stocks Canton-S and Lausanne-S (Riveron et al., 2009); an *orco* null mutant (w[*]); w[+*] orco[2] (BSC, donated by L. Vosshall, Rockefeller University, New York, USA); *orco-Gal4* (w*; P{orco-GAL4.W}11.17; TM2/TM6B, Tb1, donated by Vosshall), modified by the substitution of the third chromosome with the wildtype chromosome of the *w[1118]* stock of the Exelixis collection (Harvard, USA); *UAS-OrcoRNAi*; and line 60000, which corresponds to the *w[1118]* isogenic host strain for the RNAi library, (VDRC, Vienna).

### RNA extraction and microarrays

Four independent replicate tests were performed for each temperature regime (heat or control), and the tests were processed in different batches. In each batch, we included samples from each of the groups to randomize factors other than the applied treatment that may affect gene expression. The third antennal segments were obtained by freezing flies in liquid nitrogen, followed by fracturing and specimen collection. A large number of samples (approximately 4,000 third antennal segments for each replicate) was collected to achieve a sufficient representation of genes with low expression levels that could be missed using other protocols. Each replicate contained 2 groups of 2,000 segments collected from 11:00 to 14:00 and 16:00 to 19:00 to prevent the misassignment of gene expression due to circadian fluctuations.

Total RNA was purified with the Nucleospin RNA II kit (Macherey-Nagel), following the manufacturer’s instructions. The GeneChip® Drosophila Genome 2.0 Array developed by Affymetrix (Santa Clara, CA, USA) was used in the analyses. This array contains 18,800 probe sets, corresponding to over 13500 *D. melanogaster* genes. RNA processing, hybridization and scanning were performed at the Laboratory of Molecular Oncology, HUCA (Asturias, Spain), according the manufacturer’s instructions, from 3 μg of total RNA for each of the 8 samples.

Normalization, filtering and statistical analysis were conducted. Each Affymetrix GeneChip® probe set contains 8 to 16 paired perfect match (PM) and mismatch (MM) 25-mer probes, which are used to determine whether a given gene is expressed and to measure the expression level (signal) [55]. The Affymetrix Microarray Suite version 5 (MAS5) algorithm uses the probe-pair data to calculate the detection calls. MAS5 employs a non-parametric statistical test (Wilcoxon signed rank test) of whether significantly more perfect matches show a stronger hybridization signal than their corresponding mismatches to produce the detection call Absent (A) or Present (P) [56].

Moreover, for each probe, we set the signal intensity to reflect the relative expression of the related transcript. In this study, only probe sets with detectable expression (P) in all four replicates of the analyzed group (heat or control) were considered “Present” in that group.

The complete microarrays were used to obtain general information on the gene expression levels present in the third antennal segment and for the analysis of GO groups among the 100 genes showing the most significant changes associated with heat treatment. For further analysis, 389 genes previously related to thermal changes and olfactory reception according to current functional annotations of the *Drosophila* gene set were selected. Only these data were analyzed in detail, as for the custom-made microarrays [12].

The data were GC-RMA normalized with Bioconductor and the R project for statistical computing [57] using the BRB-Tools program developed at the Biometric Research Branch of the Division of Cancer Treatment & Diagnosis of the National Cancer Institute (Maryland, USA).

The probe sets were compared between groups of arrays using t-tests for each probe set independently, based on the normalized log-ratios for the cDNA arrays and the normalized log-intensities for the one-color oligonucleotide arrays. In the analysis, the probabilistic p-value and false discovery rate (FDR) [58] were obtained. Regarding the FDR values, an increase and/or decrease in a given gene’s expression level was considered significant if the associated value was equal to or lower than 0.1. To analyze the changes in the expression intensity of a given probe set between the control and temperature-treated groups, fold-change values were also obtained. Because the modulation due to temperature was not expected to produce extreme expression fold changes, we used the FDR 0.1 value to limit type II error (not recognizing a true effect as significant). This setting is common when microarray analysis is applied to specific tissues or cell cultures [59-61].

### RT-PCR

Total RNA from the third antennal segments of the flies in the heat-treated and control groups was isolated as described previously, and first strand cDNA was synthesized from the total amount of isolated RNA using the SuperScript first-strand synthesis system for reverse transcriptase-PCR (Invitrogen) with random primers.

Real-time PCR was performed in the 7900HT Fast Real-Time PCR System (Applied Biosystems), and cycle thresholds (CTs) were determined using SDS 2.3 software (Applied Biosystems). Each reaction was carried out with 3 replicates for each pair of primers and each condition in a final volume of 15 μl using SYBR Green® Master Mix (Applied Biosystems).

Primers (Table 7) were designed to join the end of one exon with the beginning of the next exon and were previously tested in silico for their ability to amplify cDNA products and not genomic DNA. Each pair of primers was tested together with the pair corresponding to *βtubulin60D*, which was used as a control to ensure an equal amplification efficiency by comparing the slopes of the obtained standard curves. Data analysis was carried out following the ΔΔCT method [62].

**Table 7:** Primer sequences for the RT-PCR validation experiment

**Gene**	**Forward primer**	**Reverse primer**
*gr21a*	CTGCTTCTGCAGCTTGTGGT	TAAGTGGCGATGATCGTTGT
*hsc70Cb*	ATCGGTCAGACGACAAGGAG	CGAAGGCCACAAAGGAGG
*hsp67Bb*	CCAGCATCAATATACCCTTGG	TGGCAATTTTCTCTGCTTCC
*ir21a*	CCTTCACCGGAAAATACTCG	CTCCCAGCCACCATTGTTC
*obp49a*	GTCGGGTTCTGCCTGAATG	GGCGGAGTCATGTCAAACTT
*or47b*	TGAGCCACCTCCACTGACTG	GGCCAGTTTCTCCCTGATTT
*pbprp4*	AGTTTCATAACGTCGATGACCA	TCATCAAACCGGACTTTTCG
*per*	GATCAAGAGCAGCTACAAGGTTC	GAGGTCCTCGTGGTGGTAGA
*ugt86Da*	AACACCGATCTGGTGGGAT	GCGCGGCAGATAGAAGTAGT
*βtubulin60D*	ACCAAATCGGCGCTAAGTTC	GTACTTGCCACCCGACGAC

Correspondence between the microarray and the q-PCR data for the same 9 genes was analyzed by regression line and correlation value. Statistical significance was determined by comparing regression and residual variances using an ANOVA test.

### Olfactory behavior, T-maze

A T-maze [63,64] was used to test olfactory behavior. This assay is performed in complete darkness and is a double-choice olfactory preference test. Briefly, 30 flies from the experimental or control groups were starved for 24 hours before the test. They were then introduced into a central chamber in a sliding vertical plate from the starting compartment. Once the plate was placed in the bottom position, the flies could choose between the left and right sides, containing the odorant tube and the control tube, during the 1-minute experiment. The odorant tube contained a piece of filter paper soaked with 0.5 ml of ethanol at a certain concentration, and the other side contained the solvent, water.

The olfactory index (OI) was calculated as the number of flies in the stimulus tube divided by the total number of flies at either end. The OI values ranged from 0 (maximum repulsion) to 1 (maximum attraction), with the threshold of indifference being 0.5.

In our experiments, the olfactory stimulus was ethanol (Merck, Darmstadt, Germany) diluted in water. The odorant concentration for each experiment was chosen based on the ability of a given concentration to evoke intermediate repellent responses. This is the region of the dose–response curve that gave us a maximal resolution in distinguishing sensitivity differences associated with heat shock in a previous study [6]. This concentration was 10^-1.5^ (vol/vol) in the experiment involving flies showing reduced expression of the *orco* gene due to the effect of RNAi in heterozygous *orco-Gal4/UAS-orcoRNAi* flies and 10^-0.5^ in the experiment with the *orco* null mutant.

More than 30 replicate tests were performed for each line and condition; the exact number in each case is indicated in the Results section. Statistical significance was determined using Student’s t-test.

## Abbreviations

CSDP: Cold shock domain protein; CYP: Cytochrome P-450 mono-oxygenase; FDR: False discovery rate; GR: Taste receptor; GST: Glutathione-S-transferase; HSP: Heat shock protein; IR: Variant ionotropic chemosensory receptor; OBP: Odorant binding protein; OR: Olfactory receptor; ORN: Olfactory receptor neuron; UGT: UDP-Glucuronosyl transferase

## Competing interests

The authors declare that they have no competing interests.

## Authors’ contributions

JR performed the microarray experiments and analysis and contributed to the experimental design and preparation of the manuscript. TB designed and performed the quantitative-PCR validation and contributed to the preparation of the manuscript. EA participated in the experimental design and analysis, directed the work and prepared the manuscript. All authors read and approved the final manuscript.

## Supplementary Material

Additional file 1: Table S1 Table listing the 80 genes that showed the greatest changes in expression in response to heat (10% of the genes that change significantly at P < 0.001), either increasing (black) or decreasing (blue). In both cases the fold change correspond to the ratio of the more abundant to the less abundant group, with or without heat treatment. Click here for file

Additional file 2: Table S2 Table listing the results for the 389 probe sets that were included in the detailed microarray analysis. The raw microarray data have been deposited in the following public database: ArrayExpress at the European Bioinformatics Institute (EBI), http://www.ebi.ac.uk/arrayexpress. MAGE-TAB Accession number: E-MTAB-1228. Click here for file
